# Lilium Tig—Palpitation, Carbo Veg.—Dysentery, Sulph.—Diarrhoea, Cauth.—Urinary Disease, with Comments

**Published:** 1881-05

**Authors:** C. Pearson

**Affiliations:** Washington, D. C.


### CLINICAL CASES, WITH COMMENTS.

Lilium tig.—Palpitation; Carbo veg.—Dysentery; Sulph.—Diarrhœa; Canth.—Urinary Disease.

BY C. PEARSON, M.D., WASHINGTON, D. C.

CASE 1.—Gentleman, aged sixty, tall and spare, naturally weak digestion, but strictly temperate, never making use of alcoholic liquors, tea, coffee or tobacco. Had at times for eight years been subject to attacks of rapid throbbing or beating of the heart. These attacks for five or six years always came during the day, but more recently at night as well; they were not frequent nor of long duration, varying in time of occurrence from one day to two months, and lasting from five to fifteen minutes. There were no premonitory symptoms of an attack, they came without warning, and left as suddenly; but while they lasted the pulse could scarcely be counted, running to a hundred and seventy in a minute, not full and strong, nor intermittent, but quick, weak, and at times almost a flutter. There was no pain or other symptom, except a weak, oppressed feeling in the chest, during the paroxysm, which was only slightly increased by lying on the left side; and a disposition to sigh, or to inflate the lungs. Many remedies had been taken during the past five or six years, but whether any of them had retarded or shortened the attacks was questionable, as they seemed to come more frequently and with greater violence. Believing a chronic gastric trouble to be the exciting cause, and without being certain that

Lili-tig. was adapted to this pathological condition, I prescribed this remedy in the 50 M. potency and for this one symptom alone, *rapid beating of the heart.* Cured the case promptly.

CASE 2.—Child ten months old, unweaned, had four teeth, others pressing hard on the gums; in cold, January weather, was taken with watery diarrhœa, which in a few days assumed a dysenteric form: stools, slime and blood, *more of the latter* than I ever recollect having seen in a patient so young; from six to ten stools in twenty-four hours; *not much pain, but much gas; little if any fever*, fretful, no disposition to notice any thing; wanted to be carried. Podop. 50 M. and Kali c. C.M. both failed. Carb. v. C.M. one powder after each evacuation effected a prompt improvement, and permanent cure in two days.

CASE 3.—Child, nearly same age and under treatment at the same time; teething, diarrhœa; yellow, mixed, undigested stools, ten to sixteen in twenty-four hours, more frequent from 7 to 11 A.M., not much pain or fever, but child very fretful and restless; unweaned, no appetite; stools *dreadfully acrid excoriating the parts.* Arsenic and Merc. Cor. 50 M., both failed. Sulph. CM. cured promptly.

COMMENTS:—The mongrels and others who have joined them in their tirades against fluxion potencies, will urge against these cases their usual stale arguments. 1. "The patient's imagination" (little baby's imagination!) or, 2. "infinitesimal portions of drug substance that happen not to have been washed out of the fluxion apparatus and are analogous to particles found in the low Hahnemannian preparations. Fluxion potencies are therefore shams."

"A Daniel come to judgment! Yea, a Daniel." What became of the "infinitesimal particles of drug substance" in many of these preparations, which were made up to the 30th or 200th, according to Hahnemann's formula *before* they were put into the potentizer at all? Does this fluxion process make them lower? And then I have added one hundred drops of alcohol to many of my vials when they got low, at least one dozen times in the past ten years, yet they now act as promptly as before. If they were low at first, they can not be very crude by this time; it is therefore useless to talk of their being low. They may not be quite so high as those who prepare them claim, and yet the proportion of drug matter to the vehicle is very nearly the same. No one who has ever used the low potencies for a number of years, and afterward the high as long, will fail to observe the superiority of the latter; neither will they doubt the fact that they will find their mortality list diminished more than one-half. If the physician's highest duty is to heal the sick, then let us lay aside our prejudice about "fluxion," "croton water," "bottle washing," and all such bosh, and come down to facts and figures. For my own part, though I have certainly been reasonably busy, I have rarely prescribed any thing but what purported to be 1 M. and upward (never any thing even so low as this if I had it higher) and yet I have not lost a single case of any acute disease for two years—all without a wash, gargle, plaster or poultice.

Now gentlemen! we want no fooling. No long rows of figures about "drug matter," no learned talk of pathology or the microscope. We want your statistics, give us your death list, and if you can show success, equal to this according to the number of patients treated, even then ours is still the better practice of the two, better for the patient, better for the physician, and better for homœopathy. A better showing we know you can not make, as we have been there and know whereof we speak. Besides, these fluxion potencies not only act vigorously, but in some instances give as much evidence of aggravations as can be observed from the lower, as the following case will show.

Patient, a male, aged sixty-five, chronic catarrh of the bladder of years' standing; burning and smarting pain along whole course of urethra on urinating, urine normal in quantity, but emitting a very strong, offensive smell, and depositing, to every pint, after standing a thick, ropy, gelatinous mucus, one-half inch in thickness, and so tenacious that it would not leave the vessel with the urine, and when scraped out fell in ropes, or strings two or three feet long; the color was a little darker than gum-arabic, and very nearly that of the urine. He had been greatly troubled with irregular chills of sometimes an hour's duration followed by high fever and perspiration. Gave, March 1st, Canth. M.M. 8 powders to be taken night and morning and Sac. Lac. during the day. On the 5th I received the following note which I copy verbatim, italics and all.

March 5th, 1881.

Dear Doctor,—A part of the time since you were here the urine has been entirely free from mucus, but for the last thirty-six hours it has appeared and is quite as *tough* as ever. There has been much more pain than usual in voiding the water, a sensation as if it were almost *boiling*, which continues for some time after it ceases to flow; the pain this morning is *very severe* while and after urinating more so than ever before. The last powder taken this morning. Yours, etc.

Prescribed Sac. Lac. and in one week received the following report:

March 12th, 1881.

Dear Doctor,—Since my last report there has been no appearance of mucus in the vessel, the urine is of about the proper color with *very little odor*, not much pain in emitting it, some soreness in the urethra, particularly immediately in front of the anus; tendency to void urine every two or three hours. Appetite and digestion good, sleep well, bowels in good condition, inclination to take exercise in open air whenever the weather permits. The last medicine was just the thing. Yours, etc.

He of course got more of the same, Sac. Lac. Now what does his first note indicate? We have here the pathogenetic effects of Cantharis as plainly stated as in any published proving of that medicine. The patient did not know what he was taking; it is idle to say he had not sense enough to know when it hurt him to urinate or when it did not. He called at my office to-day (20th March) more than a mile from his home, looking so well that I did not at first recognize him. We talk of patients not having faith in homœopathy, but if they had no more than a majority of physicians themselves have, most of us would have to resort to some other vocation. For patients have confidence enough to try the treatment, while physicians go on crying "humbug," fraud and imagination all their lives, never having confidence to try pure homœopathy without which they remain in blissful ignorance as to its wonderful healing powers.

